# Therapeutic Strategies for Regulating Mitochondrial Oxidative Stress

**DOI:** 10.3390/biom10010083

**Published:** 2020-01-05

**Authors:** Yuma Yamada, Yuta Takano, Satrialdi, Jiro Abe, Mitsue Hibino, Hideyoshi Harashima

**Affiliations:** 1 Faculty of Pharmaceutical Sciences, Hokkaido University, Kita-12, Nishi-6, Kita-ku, Sapporo 060-0812, Japan; satrialdi@eis.hokudai.ac.jp (S.); bm0616@eis.hokudai.ac.jp (M.H.); harasima@pharm.hokudai.ac.jp (H.H.); 2 Research Institute for Electronic Science, Hokkaido University, Kita-20 Nishi-10, Kita-ku, Sapporo 001-0020, Japan; tak@es.hokudai.ac.jp; 3 School of Pharmacy, Institut Teknologi Bandung, Ganesha 10, Bandung 40132, Indonesia; 4 Department of Pediatrics, Graduate School of Medicine, Hokkaido University, Kita-15, Nishi 7, Kita-ku, Sapporo 060-8638, Japan; jiroplus5311@med.hokudai.ac.jp

**Keywords:** mitochondria, oxidative stress, photodynamic therapy, cancer therapy, antioxidant therapy, cell therapy, drug delivery system, liposomes, MITO-Porter

## Abstract

There have been many reports on the relationship between mitochondrial oxidative stress and various types of diseases. This review covers mitochondrial targeting photodynamic therapy and photothermal therapy as a therapeutic strategy for inducing mitochondrial oxidative stress. We also discuss other mitochondrial targeting phototherapeutic methods. In addition, we discuss anti-oxidant therapy by a mitochondrial drug delivery system (DDS) as a therapeutic strategy for suppressing oxidative stress. We also describe cell therapy for reducing oxidative stress in mitochondria. Finally, we discuss the possibilities and problems associated with clinical applications of mitochondrial DDS to regulate mitochondrial oxidative stress.

## 1. Introduction

Reactive oxygen species (ROS) have several critical functions in cell signaling pathways that are involved in regulating several biological and physiological processes. ROS are natural by-products of cell metabolism, and are mainly produced through oxidative phosphorylation in mitochondria. Cell typically have a defense mechanism to eliminate ROS and control ROS levels at safe concentrations through antioxidant systems. These biological defense systems can be classified into enzyme-based systems, such as superoxide dismutase (SOD), glutathione (GSH), peroxiredoxins, and the non-enzyme-based systems including several vitamins, polyphenols, and coenzyme Q_10_ (CoQ_10_) [1]. The disproportion between ROS production and ROS scavenging capacity results in oxidative stress, leading to mitochondrial dysfunction. It further contributes to the acceleration of several disorders such as age-related neurodegenerative diseases, diabetes and its complications, various types of cancer, and several lung diseases [2,3,4]. Therefore, a therapeutic strategy that allows mitochondria oxidative levels to be regulated, either by suppressing or promoting ROS production, could be greatly promising.

Antioxidant therapy is a promising strategy for controlling the overproduction of ROS. This approach has been widely adopted for the treatment of a number of mitochondrial dysfunction-related diseases and have shown promising outcomes. For example, the neuroprotective effect of antioxidants could significantly suppress the progression of neuronal cell damage, particularly in the case of Parkinson’s and Alzheimer’s diseases, by the direct neutralization of ROS and by suppressing inflammation [5,6]. Furthermore, CoQ_10_, a potent ubiquinone antioxidant, has been reported to show significant protective effects on mitochondria of the pancreatic beta cells during the oxidative stress induced by the chronic use of tacrolimus [7]. Due to the highly hydrophobic characteristics and the fact that most of the antioxidant molecules accumulate in a nonspecific manner, a high dose is needed to obtain the desired effects. Thus, the selective delivery of this compound would be expected to further strengthen its effectivity.

In the context of cancer therapy, mitochondrial ROS were found to play an important role as a signaling molecule in controlling cell proliferation by virtue of its ability to regulate the mitogen-activated protein kinase (MAPK)/extracellular signal-regulated kinase (ERK) signaling pathway [8]. Moreover, antioxidant activity, such as GSH and thioredoxin, was also reported to be essential for the initiation of and the progression of cancer. Inhibition of the antioxidant activity resulted in a significant reduction in tumor progression and metastasis [9,10,11]. These findings suggest that cancer cells are maintained under conditions of ROS stress and that antioxidant supplementation may be irrelevant for cancer therapy [12,13,14]. However, ROS interact with several major biologically active molecules such as proteins, lipids, and nucleic acids, leading to irreversible oxidative damage and the further induction of lethal effects for the cells. Therefore, promoting ROS production could be a promising strategy for treating tumors. There are numerous methods that can be employed to promote ROS levels in cancers, i.e., depleting or inhibiting antioxidant activity [15,16] and inhibiting the mitochondrial respiratory system [17], and producing an excessive amount of ROS through photochemical reactions. The latter effort shows a high selectivity for tumor cells, making it more encouraging in contrast to other efforts.

**Figure 1:**
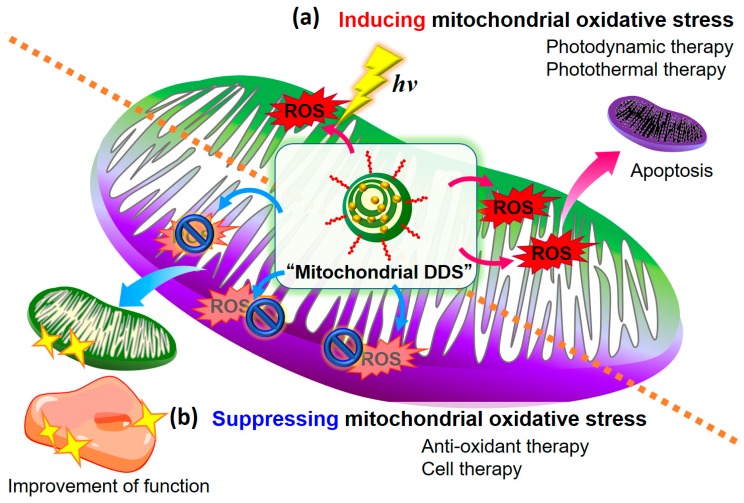
Schematic illustration of therapeutic strategies for regulating mitochondrial oxidative stress. Given that mitochondria are capable of producing reactive oxygen species (ROS) they are a source of oxidative stress. It follows that there are two types of therapeutic strategies for inducing and suppressing oxidative stress, which can be broadly divided into two main therapies. (**a**) Photodynamic and photothermal therapies by the mitochondrial delivery of photosensitizers that induce oxidative stress, thus leading to cell death. (**b**) Anti-oxidant therapy and cell therapy that deliver therapeutic molecules that suppress mitochondrial oxidative stress to mitochondria, removing ROS and improving mitochondrial and cellular functions.

This review focuses on therapeutic strategies for regulating mitochondrial oxidative stress. The main theme includes therapeutic strategies designed to induce oxidative stress and suppress mitochondrial oxidative stress (Figure 1). As therapeutic strategies to induce mitochondrial oxidative stress, we introduce mitochondrial targeting photodynamic therapy (PDT) and other mitochondrial targeting phototherapeutic methods. As a strategy to suppress mitochondrial oxidative stress, we discuss the use of a mitochondrial drug delivery system (DDS) for anti-oxidant therapy. We also introduce cell therapy to reduce oxidative stress in mitochondria. Moreover, we outline the therapeutic strategy based on the use of a MITO-Porter that was developed in our laboratory to control mitochondrial oxidative stress [18,19,20,21,22].

## 2. Therapeutic Strategy to Induce Oxidative Stress

### 2.1. Mitochondrial Targeting PDT for Cancer Therapy

**Figure 2:**
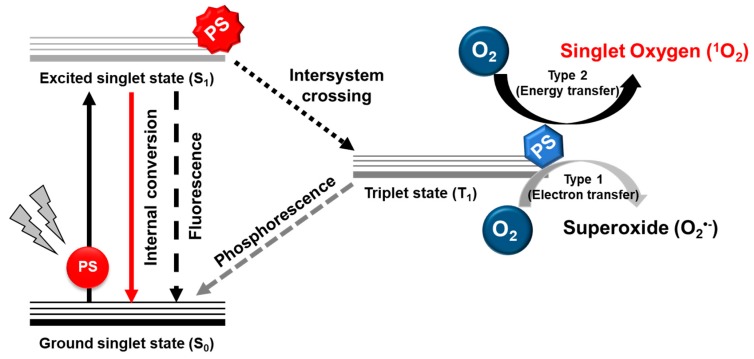
Schematic illustration of the mechanism behind the photodynamic process. The PS harvests energy from light with a suitable wavelength to excite an electron from the ground singlet state (S_0_) to the excited singlet state (S_1_) followed by intersystem crossing to the triplet state (T_1_). In this state, PS interacts with oxygen molecule to generate ROS.

One of the most attractive approaches for amplifying ROS levels is through an energy transfer reactions mediated by light-activated molecules (photosensitizers), a therapy known as photodynamic therapy (PDT) [23]. This innovative strategy offers high selectivity towards tumors while having minimal effects on healthy cells. The PDT effect originates from the dynamic interaction between a photosensitizer (PS), light, and oxygen molecules, leading to the production of the lethal level of ROS, primarily highly reactive singlet oxygen (^1^O_2_) (Figure 2). The maximum PDT effect may be derived from the specific location of the photochemical reaction inside the tumor cells. It is also reinforced by the nature of ^1^O_2_, which has a very short lifetime (~48 ns) and limited diffusion capacity (~20 nm) [24]. Therefore, the specific delivery of PS, particularly at the subcellular level, is one of the most critical aspects to be considered for optimizing the effectiveness of PDT. The distinction in the subcellular localization of the PS constitutes a different cell death mechanism. For example, mitochondria accumulation could initiate the apoptosis pathway, while plasma membrane localization may provoke the necrotic cell death [25,26]. From the viewpoint of cancer therapy, apoptosis cell death is preferable due to the negligible inflammation effects, which would lessen the side effects on healthy cells.

**Figure 3:**
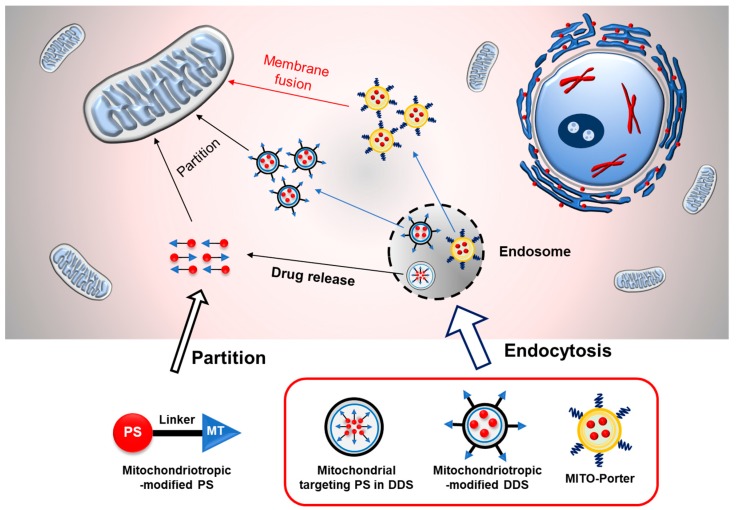
Conceptual illustration of the cellular internalization and mitochondrial delivery mechanism of several strategies in mitochondrial targeting PDT. Mitochondriotropic-modified PS would be taken up by cells through the partition process, while the nanoparticle-based strategy largely results in internalization via the endocytic pathway followed by endosomal escape. Electrostatic interactions with the negatively-charged mitochondria membrane promotes the delivery of the PS to mitochondria by partition or a membrane fusion process.

Oxygen is a critical component that must be present during the photochemical process of PDT. The lack of oxygen (hypoxia) in some solid tumors can result in a decrease or even the loss of the effectiveness of PDT. It has been reported that the inhibition of the mitochondrial electron transport chain results in a significant increase in the oxygen concentration in the mitochondrial compartment Ref [27], which could be an advantage for PDT applications. A more recent report suggested that mitochondrial targeting PDT could inhibit mitochondrial respiration, leading to a change in intramitochondrial oxygen levels, and further enhancing the PDT effect under hypoxic conditions Ref [28]. In addition, the overproduction of ROS in mitochondria during the PDT process causes the depolarization of the mitochondria membrane, resulting in the release of proapoptotic proteins, particularly cytochrome c, from the inner mitochondrial membrane to the cytosol, further activating apoptosis cell death [25,29,30]. These findings imply the importance of mitochondrial targeting in PDT applications. Therefore, several creative and innovative approaches have been developed regarding the mitochondrial targeting of PDT, ranging from the chemical conjugation of the PS with the mitochondrial targeting moiety to the utilization of a drug delivery system. In the following section, we discuss some of the more fascinating approaches in mitochondrial targeting PDT. The conceptual illustration is shown in Figure 3. A mitochondrial targeting PDT strategy for cancer therapy is summarized in Table 1.

#### 2.1.1. Chemical Conjugation of the PS with Mitochondrial Targeting Ligand

Several mitochondrial targeting ligands or mitochondriotropics have been used to modify the currently existing or new generation of PS to achieve selective accumulation in the mitochondrial compartment of tumors. One of the most popular mitochondriotropics that has been widely used for mitochondrial delivery is triphenylphosphonium (TPP). It possesses an excellent ability to intensify both cellular uptake efficiency and mitochondrial accumulation [31]. Since the TPP group has a stable and appropriate lipophilicity and cationic charge, it is a powerful targeting moiety to mitochondria which possess a highly negative membrane potential (ca. −150 to −170 mV) [32]. Verteporfin, an FDA-approved PS, has been conjugated with several mitochondrial targeting ligands i.e., TPP, guanidinium, and bisguanidinium which resulted in different levels of mitochondrial accumulation. Interestingly, the TPP derivative exhibited the highest mitochondrial accumulation level and the effect is correlated with the improvement in PDT cytotoxicity as opposed to the other two derivatives and its parent compound [25]. Furthermore, the conjugation of TPP into the octahedral molybdenum, which has a robust ability to produce ^1^O_2_, leads to an improvement in cellular uptake efficiency and mitochondrial accumulation. This further improved the PDT killing ability, as indicated by the low minimum required concentration to eliminate half of the cell population (EC_50_) of approximately 0.1 μM [33]. However, 460-nm light is needed to activate this system, which is not ideal for clinical applications due to its poor tissue penetration ability.

The utilization of the other mitochondrial targeting ligands has been reported as an alternative approach to the use of TPP. Phthalocyanine PS can be actively delivered to the mitochondrial compartment of human nasopharyngeal carcinoma (HK-1) cells by being axially ligated with rhodamine B, which effectively induces apoptotic cell death [34]. Moreover, the chemical conjugation of protoporphyrin IX (PpIX) with a mitochondrial targeting peptide, namely (KLAKLAK)_2_, strongly induced in situ ^1^O_2_ generation in the mitochondrial compartment, leading to an enhanced PDT killing capacity both in vitro and in vivo. Photochemical internalization has been employed to improve cellular uptake efficiency by a short irradiation process to induce the peroxidation of membrane lipid, leading to the alteration of membrane permeability [35]. Also, the conjugation of another phthalocyanine PS, IR700DX, with a specific ligand of the mitochondrial outer membrane translocator protein (TSPO), showed a significant increase in the mitochondrial accumulation level and led to the activation of the apoptotic pathway. This system is highly effective for treating tumors that overexpress mitochondrial TSPO, such as some types of breast, colorectal, prostate, and brain cancers, while it is ineffective in TSPO-negative cancer cells [36].

The chemical conjugation of the PS with a mitochondriotropic agent could be a promising strategy for delivering a wide range of PSs that are selective for the mitochondrial compartments of tumors. Moreover, this strategy provides a good translatability towards mass production, which is one of the most critical issues for clinical applications. However, conjugation with the mitochondriotropic agent will not significantly affect the hydrophobic nature of the system and the tendency for PS to form aggregates in aqueous solution, leading to difficulties in the formulation of the compound. Also, because the conjugate system lacks tumor specificity, this would lead to an adverse effect on healthy tissues.

#### 2.1.2. Mitochondrial Targeted PS in Drug Delivery System

In the last few decades, nanoparticles (NPs) have evolved as the most important drug delivery strategy due to their excellent ability to improve the in vivo stability of a wide range of molecules, leading to better tumor accumulation. NPs systems could also be applied to delivering a wide variety of PSs with better tumor specificity compared to the free-drug [37]. Moreover, it is also possible to add several targeting ligands to the surface of the NPs to further intensify their tumor-targeting ability. A promising strategy for the mitochondrial targeting of PDT involves encapsulating the mitochondrial-targeted PS in a nanocarrier system. The ability of the PS to accumulate inside mitochondria could be accomplished either by the intrinsic mitochondrial targeting ability of the PS itself or by conjugation with the mitochondriotropic, as discussed above. Poly (ethylene glycol)-*b*-poly (2-isopropyl amino) ethyl methacrylate (mPEG-*b*-PDPA), a pH-responsive copolymer, has been used to specifically deliver TPP-pyropheophorbide-a (TPPa) PS to the mitochondrial compartments of tumors. The tumor accumulation of this system is achieved by the enhanced permeability and retention (EPR) effect, followed by endocytosis. During the acidification of the endosome environment, the polymeric NPs rapidly dissociate, leading to the release of the TPPa, which could then effectively escape from the endosome and accumulate in the mitochondria [29]. A mitochondrially selective PS, namely IR-Pyr, has been used to form micellar aggregates through electrostatic interactions with negatively charged hyaluronic acid (HA) molecules to produce dual-targeting ability towards a tumor and its mitochondrial compartment. This system was highly tumor-selective, due to the overexpression of the cell surface CD44 as the HA receptor. The near-infrared light was used to activate the PS, resulting in an effective PDT killing effect with an EC_50_ value of 5–7 μM against HeLa and MDA-MB-231 cell lines [38].

The cationic porphyrin (MitoTPP) molecule, a mitochondrial targeting PS, has been incorporated into the nanographene oxide (NGO) modified with polyethylene glycol (PEG) and folic acid. This system showed a selective accumulation in the mitochondrial compartment of folate receptor-positive cells to induce the apoptotic cell death when irradiated with 650-nm light [39]. Furthermore, folate-modified cholesteryl-bovine serum albumin NPs have been used to deliver the TPP-pheophorbide-a PS into an orthotopic glioblastoma-xenografted mouse. The accumulation of this system in a brain tumor reached 0.2% of the injected dose within 2 h after administration. By using the fiber optic cannula to more precisely deliver light, this system resulted in a remarkable inhibition of tumor growth [40]. The application of the drug delivery system to delivering the mitochondria-targeted PS represents a promising and potential approach for improving PDT tumor selectivity. Because this nanocarrier system is flexible, it could be modified with a number ofl targeting ligands for specific targeting purposes. Moreover, drug release is a critical aspect to be considered because the NPs system does not have mitochondria-targeted ability. Whereas, the complicated system associated with this strategy may be an obstacle in mass production for clinical applications.

#### 2.1.3. Mitochondrial Targeting Ligand-Modified Drug Delivery System

Diverse efforts have been made regarding the use of the mitochondrial targeting ligand-modified NPs to deliver several PSs into the mitochondrial compartment of tumors. The lipid-based theranosome with a TPP surface modification was used to deliver chlorin e6 (Ce6) PS and IR780 to the mitochondrial compartment of HeLa cells. IR780 has a specific role in regulating the targeted generation of ^1^O_2_ based on the fluorescence resonance energy transfer (FRET) effect with Ce6, also, to induce a photothermal effect by irradiation with 808 nm light. The ^1^O_2_ generation ability of the Ce6 is rescued after the photoinduced degradation of IR780, which further provokes significant PDT toxicity compared to the non-mitochondrial targeting counterpart [41].

The utilization of the upconversion nanoparticles (UCNPs) could also be a promising strategy for reaching deep-seated tumors with negligible interactions with endogenous molecules [42]. This system can up-convert two or more low-level energy photons (near-infrared light) into one high-energy photon (ultraviolet-visible light) [43]. The high-energy photons emitted by the UCNPs, during the deep penetrating light irradiation, could be the energy source needed to activate the photosensitizer and induce the production of ROS [44]. The surface modification of UCNPs carrying titanium dioxide (TiO_2_) with TPP leads to the selective accumulation in mitochondria and cause a mitochondrial ROS burst during irradiation with a 980-nm laser. This system has shown significant antitumor activity in the case of an MCF-7-bearing mouse model [30].

Furthermore, the mitochondrial delivery of hybrid nanoparticles containing UCNPs and graphene quantum dots (GQD) has been achieved by decorating the nanoparticles with tetramethylrhodamine-5-isothiocyanate (TRITC). The mitochondrial targeting PDT of this system efficiently inhibited tumor growth by 75.3% in comparison to non-mitochondrial targeting with an inhibition rate of 70.2% in a 4T1 tumor xenograft model [45]. Hybridization of the UCNPs and a polymeric system was reported to be an effective nanocarrier for delivering pheophorbide a (Ppa) PS. The hybrid UCNPs-polymer was further modified with the trans-activating transcriptional activator (TAT) peptide, an arginine-rich cell-penetrating peptide, which has a dual function in enhancing cellular uptake efficiency and simultaneously targeting mitochondria. The TAT modification showed a significant improvement in cellular uptake and mitochondrial targeting efficiency, leading to an increase in PDT killing capacity [46].

The involvement of a drug delivery system, particularly NPs, in the PDT field has opened new innovative research opportunities due to the encouraging features of the NPs. The NPs can be modified with several targeting ligands to manipulate the pharmacokinetic profile of the PS, thus leading to an enhanced tumor accumulation. Moreover, NPs could be used to encapsulate a large amount of the hydrophobic PS, permitting systemic administration with a negligible risk of embolisms. However, unwanted drug release during the internalization process could be a negative effect on the level of mitochondrial accumulation due to the lack of mitochondrial targeting ability of the PS.

#### 2.1.4. Validation of Cancer Therapeutic Strategy Using MITO-Porter System

Since 2008, our group has made extensive efforts to develop a smart mitochondrial targeting liposomal-based nanocarrier, namely a MITO-Porter system. The delivery of this system to mitochondria is achieved through electrostatic interactions and fusion with the mitochondrial membrane [18]. The combination of a highly fusogenic lipid, 1,2-dioleoyl-sn-glycero-3-phosphatidyl ethanolamine (DOPE) and a cell-penetrating peptide, octaarginine (R8) plays an essential role in the cell internalization process via macropinocytosis, endosomal escape, and mitochondrial accumulation [19]. For mitochondrial targeting cancer therapy, the MITO-Porter system has been used to significantly improve the antitumor activity of an aminoglycoside drug against HeLa cells and an anthracycline compound toward doxorubicin (DOX) -resistant renal cancer cells [47,48]. We are currently developing a novel mitochondrial targeting PDT system consisting of a newly synthesized porphyrin-type PS, namely rTPA, that will be used in combination with the MITO-Porter system. The synergistic action of the rTPA compound and the MITO-Porter system could effectively induce apoptotic cell death during 700-nm light irradiation with a very low EC_50_ value of 0.16 μM and 0.41 μM against HeLa and SAS cells, respectively [49]. These findings suggest that the unique mitochondrial targeting strategy of MITO-Porter system represents a promising strategy for improving PDT outcomes.

### 2.2. Other Phototherapies Targeting Mitochondria

The essential roles of mitochondria for viability inspire various approaches to phototherapy not only by the PDT effect, as described above. In this section, other phototherapeoutic approaches, such as photothermal therapy (PTT), a photo-uncaging strategy, and photoinduced electron transfer reactions to trigger oxidative stress in mitochondria are reviewed. The mitochondrial targeting PTT strategy for cancer therapy is summarized in Table 1.

#### 2.2.1. Mitochondria Targeted Photothermal Therapy

PTT is a class of phototherapies that have been intensively studied to date. PTT utilizes the heat generated by photoirradiation to increase the temperature in cells [53,54]. Proteins in the cells undergo denaturation and the cell membrane is disrupted during the localized application of heat, which gradually leads to cell death. The thermal localized PTT comprises the hyperthermia region (41 °C–48 °C) to the region of irreversible injury (48 °C–60 °C). The application of a temperature exceedig 60 °C leads to rapid protein coagulation and drastic necrosis activation, resulting in cell death. Among the cellular compartments, mitochondria are particularly sensitive to an increase in temperature [55,56]. Therefore, a PTT strategy for targeting mitochondria is often used for either photoactivating or photokilling cells.

The use of low-level laser (light) therapy (LLLT) to stimulate cells has been extensively studied [57]. Several mechanisms of action have been proposed to date in attempts to explain the effects of LLLT on mitochondria. Although the mechanism responsible has not been completely revealed, it is generally thought that the process relies on an induced thermal effect or the activation of cytochrome-*c* oxidase by illumination [58]. Low-level laser radiation increases enzymatic activities which may be triggered by the generation of ROS caused by both PDT and PTT from the intrinsic molecules in mitochondria.

**Figure 4:**
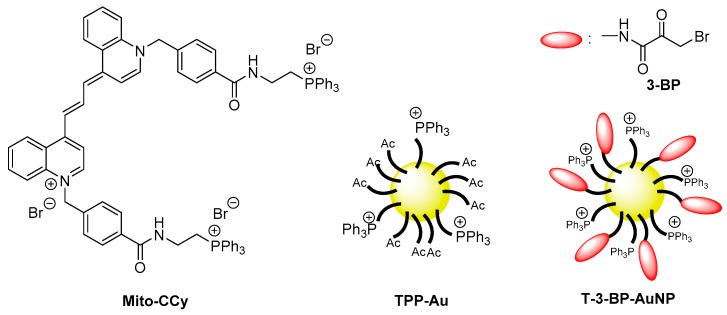
Mitochondria-targeting PTT reagents. Mito-CCy is a molecular based PTT reagent. TPP-Au is a gold nanoparticle-based PTT reagent, and T-3-BP-AuNP is combined with chemotherapy reagent 3-BP.

In contrast to LLLT, high-level laser radiation induces cell death which is probably due to the generation of excess ROS which cannot be suppressed by enzymatic activities. The use of a high-level laser increases not only the direct generation of ROS but also the temperature which can cause apoptosis by elevating the generation of ROS by mitochondria. In this context, functional molecules possessing a high efficiency for photothermal energy conversion have been studied in attempts to utilize PTT for cancer treatment. As an example, Jung and co-workers reported on the use of cryptocyanine-based molecules combined with a mitochondrial-targeting moiety, TPP (Mito-CCy, Figure 4) [50]. It was demonstrated that Mito-CCy was capable of both mitochondria-targeting and NIR-absorbing properties. Laser irradiation (730 nm, 2.3 W/cm^2^) of Mito-CCy increased the temperature of a DMSO solution by 13.5 °C within 5 min. Mito-CCy showed a highly efficient photothermal conversion in mitochondria resulting in an efficient cancer killing effect on HeLa cells. It was confirmed that ROS was not directly generated upon the laser irradiation. However, in the presence of inhibitors of the mitochondrial respiratory chain complexes I and III, ROS generation and the resulting cencer killing effect was suppressed. This result indicates that PTT-mediated ROS generation in mitochondria requires an electron transport chain that functions in whole or in part for of the photothermal effect of Mito-CCy.

Carbon nanomaterials have been developed into promising agents for PTT. The inherent biocompatibility of carbon-based materials strengthened the applicability of these materials for PTT. SWNTs functionalized with PL-PEG and FITC were developed and found to be located exclusively in mitochondria in tumor and normal cells due to the mitochondrial transmembrane potential [59]. The functionalized SWNTs showed mitochondria-targeting and a PTT effect in mitochondria [60]. Surprisingly, using a laser with a quite long wavelength to irradiate SWNTs in the NIR region (980-nm) selectively killed cancer cells via mitochondrial damage. SWNTs acted efficiently to convert 980-nm laser energy into heat and selectively destroyed the target mitochondria, resulting in mitochondrial depolarization, cytochrome c release, and caspase 3 activation. The use of a combination of a NIR-laser and SWNTs showed an impressive efficiency in suppressing tumor growth in a model of breast cancer, and in some cases resulted in the complete regression of the tumor.

Regarding clinical applications of PTT, undesired damage to adjacent normal tissue must be avoided. Ma and co-workers invented a spherical gold nanoparticle that could be used to selectively target cancerous mitochondria (TPP-Au, Figure 4) [51]. The TPP moiety in TPP-Au permitted the nanoparticles to preferentially accumulated inside tumor mitochondria. Because the cellular membrane and the mitochondrial membrane potentials are different between cancer cells and normal cells, being more highly negative in cancer cells than those in normal cells [61], TPP-Au accumulates differently between cancer tissue and normal tissue, which can avoid undesired PTT effects on adjacent normal tissues. As the result, TPP-Au can selectively reach the threshold to activate the interparticle plasmonic coupling effect among gold nanoparticles in cancer cells, but not in normal cells, resulting in selective light-thermal conversion and tumor mitochondrial dysfunction. An in vivo study showed that the temperature increase in irradiated tumor tissue was almost four times that in adjacent normal tissue.

PTT can be paired with chemotherapy drugs such as 3-bromopyruvate (3-BP). 3-BP is an alkylating agent that inhibits HK2 and selectively kills cancer cells [62], and is capable of suppressing tumor cell glycolytic capacity by abolishing mitochondrial-bound HK2 activity [63]. 3-BP is also involved in the inhibition of the other mitochondrial functions such as succinate dehydrogenase, a phosphate carrier, and an adenine nucleotide carrier [52]. A mitochondria-targeted gold nanoparticle (T-3-BP-AuNP, Figure 4) [52], decorated with 3-BP and TPP was designed and synthesized for use in the delivery of 3-BP to cancer cell mitochondria by taking the advantage of the higher mitochondrial membrane potential in cancer cells compared to that in normal cells. The results showed that the 3-BP mitochondrial compartmentalization is time-dependent when used with a T-AuNP system and involves different 3-BP targets in the mitochondrial compartments, further boosting 3-BP function. When T-3-BP-AuNP was illuminated with a 660 nm light, the T-3-BP-AuNP generated heat. In addition to the PTT effect, the generated heat was used to release 3-BP specifically to mitochondria and a subsequent laser irradiation further enhanced the therapeutic efficiency for killing PC3 and DU145 cell lines, but did not show any significant toxicity in normal hMSC cells.

#### 2.2.2. Photouncaging Strategy Targetting Mitochondria

As discussed above, a photostimulation strategy can be used for releasing a drug and it can control ROS levels in mitochondria. Mitochondria-responsive drug release along with heat-shock was developed as an approach using light for activating the lipophilic agent IR-780-modified glycolipid conjugates (CSOSA) (IR780-CSOSA) combined with DOX [64]. When IR780-CSOSA is subjected to laser irradiation, the resulting photothermal conversion can weaken the hydrophobic association between the center of the micelles and DOX, thus triggering spontaneous micellular swelling which releases DOX into mitochondria for ROS amplification. It was found that the photoconversion can also induce mitochondrial-specific heat-shock to induce the rapid production of ROS to kill cancer cells more efficiently.

**Figure 5:**
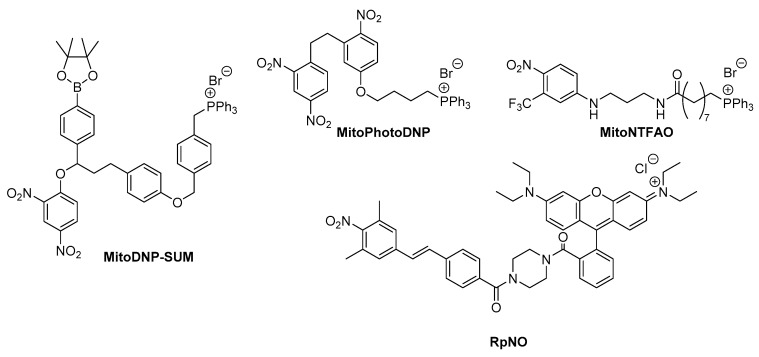
Mitochondria-targeting photoinduced nitric oxide releasing molecules. MitoDNP-SUM and MitoPhotoDNP can release DNP, and NiToNTFAO and RpNO can release NO, by photoirradiation.

Photoinduced cleavage of a molecule to release a drug molecule, also known as photo-uncaging, is a powerful method for the selective release of a drug in mitochondria. 2,4-dinitrophenol (DNP) is one of the more extensively studied uncouplers that utilizes photo-uncaging to suppress the oxidative phosphorylation, which governs the synthesis of ATP, which ultimately causes mitochondrial dysfunction. An interesting approach was proposed for mitochondria-targeted “caged” DNP, activatable by light (MitoPhotoDNP, Figure 5) [65]. MitoPhotoDNP can release a protonophore DNP within selected mitochondria by the photo-triggered uncaging of DNP. As a result, MitoPhotoDNP allows us to control or stop ATP production andcalcium ion uptake. With using a precisely controlled laser, it is possible to select a single mitochondrion or a localized subpopulation of mitochondria within a cell. A different strategy for targeting the release of DNP was reported using a trifunctional compound bearing a “caged” DNP moiety, mitochondria-targeting TPP^+^, and the H_2_O_2_ (and ONOO^−^) reactive boronate moiety for DNP activation (MitoDNP-SUM, Figure 5) [66]. The uncaging of DNP can be triggered by endogenous H_2_O_2_ in mitochondria. The compound showed permitted the selective release of uncaged DNP in mitochondria under conditions of H_2_O_2_ production.

Nitric oxide (NO) is also a good motif for creating photouncaging drugs because NO is an endogenous messenger that is ubiquitously produced by mammalian tissues and is involved in many physiological and pathophysiological processes [67]. For example, NO encourages cancer growth, whereas high levels (micromolar) decrease cancer progression [68]. To develop photouncaging drugs for NO release, Sodano and co-workers reported on a series of compounds in which *N*-[4- nitro-3-(trifluoromethyl)phenyl]propane-1,3-diamine was linked with an amide bridge to alkyltriphenyl- phosphonium chains of variable length. One of the compounds, MitoNTFAO [(9-((3-((4-nitro-3-(trifluoromethyl)phenyl)amino)propyl)amino)-9-oxononyl) triphenylphosphonium bromide] (Figure 5) displayed both the highest accumulation and the lowest toxicity towards A549 cells after the irradiation-mediated release of NO in mitochondria [69]. An alkyl chain with a suitable length with a collapsed conformation which encourages orbital overlap and stabilizes the intermediate phenoxy radical, resulted in the highly efficient photorelease of NO.

Another mitochondria-targeting NO donor in which a TPP moiety was not used was reported. The molecule consists of a photocontrollable NO release moiety of 2,6-dimethylnitrobenzene and a rhodamine moiety for mitochondrial targeting (RpNO, Figure 5) [70]. The rhodamine moiety is known to show mitochondria-targeting ability because of its moderate hydrophilicity and cationic properties. The release of NO from RpNO was confirmed by ESR analysis in aqueous solutions. DAF-FM DA, which is a NO-specific fluorescence test, confirmed the release of NO from RpNO in HCT116 colon cancer cells. The localization of RpNO in mitochondria was confirmed with MitoTracker Green FM, a mitochondrial dye, in HCT116 cells and showed a photodependent cancer-killing effect. The importance of NO was confirmed by the fact that the cytotoxicity was diminished in the presence of b-NADH and P450, which are NO reductases. This study showed that an approach using a photouncaging NO donor is an appropriate and promising mitochondria-specific phototherapy.

#### 2.2.3. Mitochondria Targeting Photoinduced Electron Transfer to Induce Redox Reactions

One inevitable drawback of PDT is that it may not be effective in hypoxic environments, which are the case in various cancer tissues because of the high requirement for and consumption of oxygen by cancer cells. In such an environment, direct photoinduced oxidation or reduction reactions are considered to be effective in causing cancer cell damage. An appropriate combination of an electron donating molecule (D) and an electron accepting molecule (A) can be used for this purpose. In the same system, photoexcitation remarkably promotes an electron transfer reaction from D to A, resulting in one-electron reduced D (D^●+^) and one-electron accepted A (A^●−^) [71].

An intermolecular electron transfer reaction following the photoinduced intramolecular charge separation in a D-A molecule was demonstrated to change the lipid structure in mitochondria. 9-Mesityl-10-methylacridinium and its derivatives were used to induce mitochondria specific lipid photo-oxidation by taking advantage of the intermolecular electron transfer reaction [72]. Mitochondria-specific localization of the molecules was accomplished by their strong interaction with anionic lipids in mitochondria owing to its cationic and π-conjugated nature. In HeLa cells, the molecules triggered mitochondrial lipid oxidation, which was followed by apoptotic cell death, under illumination within a few seconds.

Not only the oxidation reaction, but a reduction reaction is possible by using a different type of D-A molecule. A molecular system based on a fullerene-zinc porphyrin-ferrocene triad demonstrated an excellent electron-donating ability which was triggered by illumination [73]. The water-soluble triad molecule was examined in a living cell system, HeLa cells. Although, the naked molecule was found to be incapable of penetrating the outer cell membrane because of its relatively large hydrophobic moiety, a successful combination with the MITO-Porter system enabled the selective transportation of the molecule into mitochondria. It was also verified that light illumination effectively leads to the generation of superoxide, a type of ROS, in mitochondria as a result of the efficient electron transfer from molecule to oxygen. A high dose of the molecule/MITO-Porter conjugate and a long period of illumination effectively caused the death of cancer cells via photoinduced damage from significant amounts of superoxide that were present.

## 3. Therapeutic Strategy for Depressing Mitochondrial Oxidative Stress

At this point, we discussed therapeutic strategies based on the induction of mitochondrial oxidative stress. In particular, the focus was on cancer therapeutic strategies. On the other hand, a therapeutic method for suppressing mitochondrial oxidative stress can be also considered to be a useful therapeutic strategy. While the respiratory chain in mitochondria continuously produce ATP, it also supplies substantial amounts of ROS. Enzymes such as catalase and superoxide dismutase in mitochondria serve to quench ROS, which can play an important role in the physiological regulation of mitochondrial biogenesis. Excessive levels of ROS can damage lipids, proteins or nucleic acids and play a pathogenic role in various cells or tissues. Exaggerated oxidative stress from mitochondria could become worse and would rapidly expand. This is referred to as a “vicious cycle” in mitochondria biology. ROS produced by mitochondria cause oxidative damage that impairs the ability of mitochondria to carry out their metabolic functions through ATP production or cellular redox signaling pathways. Massive levels of oxidative stress finally affect human homeostasis in vital organs such as the brain, heart, or liver, thus contributing to human pathologies. We will now focus on that point in the following section including antioxidant therapy and cell therapy.

### 3.1. Antioxidant Therapy Using Mitochondrial DDS

Antioxidant supplements and drugs have been used to scavenge free radicals that are produced in mitochondria. Certain types of drugs inhibit mitochondria, thereby killing cancer cells; they protect cells from oxidative damage; and, they repair defects. Because of the complex nature of the mitochondrion, different strategies may be required for the mitochondrial uptake of different pharmacotherapeutic agents. During the last decade, there have been many reports related to the delivery of antioxidants to mitochondria, most of which involve mitochondrial delivery using TPP, DQA, SS peptides, mitochondrial targeting signal peptides and MITO-Porters [19,74]. As described above, there have been many reports that TPP is useful as mitochondrial molecule delivery device, and TPP has been also used as an antioxidant molecule. In this session, we review mitochondrial antioxidant delivery using TPP (Table 2). In addition, we introduce the delivery of antioxidant molecules using a MITO-Porter system.

#### 3.1.1. Mitochondria-Targeted Delivery of Antioxidants Using the TPP System

Selective targeting of antioxidants to mitochondria in intact cells was initially reported by Murphy and coworkers [75]. They synthesized MitoQ, a ubiquinone (coenzyme Q_10_) molecule conjugated with TPP that selectively accumulates in mitochondria [76], and many researchers have reported various antioxidant therapeutic strategies using MitoQ to date. For example, in order to verify a therapeutic effect protects against oxidative cell death (such as cerebral ischemia), MitoQ was treated with neuronal HT22 cells [77].

It was also reported that TPP-TEMPO that was synthesized by the conjugation of 2,2,6,6-tetramethylpiperidine 1-oxyl) (TEMPO) exerted an antioxidant effect and TPP [78]. Although TPP has been used for the mitochondrial delivery of small hydrophobic molecules, the mitochondrial delivery of a hydrophilic antioxidant chemical (vitamin C) modified with TPP (MitoC) could be achieved [79]. In this report, it was shown that MitoC has the ability to protect against lipid peroxidation at concentrations several hundred times lower than ascorbate. There have been many reports on the therapeutic effects of these TPP analogs due to mitochondrial targeting and their antioxidant effects. However, the hurdles for clinical applications would be high, and it appears that an antioxidant simply conjugated with a functional element is insufficient.

We introduce the concept of neuroprotective effects of mitochondria-targeted plastoquinone (SkQR1), which is a conjugate of plastoquinone with decylrhodamine 19 [80]. Plastoquinone is a major quinone contained in chloroplasts and cyanobacteria and acts as an electron transfer carrier in the photosynthesis system and exerts antioxidant effects. In a study in which neonatal hypoxic-ischemic brain injury model rats were treated with SkQR1, the infarction area of the SkQR1 treated group was reduced by about 40%, compared with that of the non-treated group. In addition, motion and neurological functions were significantly improved.

Under such a situation, it was reported that TPP-modified therapeutic drug analogs were encapsulated in nanoparticles with tissue targeting ability, the pharmacokinetics of which were controlled so as to permit the cargo to be delivered to the target site, and mitochondrial delivery was achieved by TPP at the site, followed by the appearance of a therapeutic effect. Brenza et al. reported that MitoQ was encapsulated in brain targeting nanoparticles composed of poly (lactic-co-glycolic acid) (PLGA) [Mito-Apo] and these particles accumulated in the brain [81]. Wu et al. reported that a TPP-modified free radical scavenger was encapsulated in liver targeting lipid nanoparticles [MitoPBN], after which, the anti-oxidant therapeutic effect was examined using a diabetes model animal [82]. In this study, MitoPBN achieved successful liver targeting and increased mitochondrial maximal respiration, resulting in an increase in glycolysis by 30%. This result suggests that the improvement of redox balance in mitochondria contributes to normalizing glucose metabolism in type 2 diabetes.

In addition, it was reported that a nanoparticle modified with TPP was used for the mitochondrial delivery of an antioxidant molecule. Zhang et al. reported on the development of lipid-polymeric nanocarriers (LPNs) encapsulating tanshinone IIA (TN), which exerted antioxidant effects [83]. The LPNs (TPP-TPGS/TN/LPNs) were composed of d-α-tocopheryl polyethylene glycol 1000 succinate (TPGS) linked to the TPP, and showed mitochondrial targeting activity. In a study dealing with a therapy for myocardial infarction, the administration of TPP-TPGS/TN/LPNs significantly increased the accumulation of TN in the heart and reduced the infarctional area by about 50% compared to the non-administered group. N-acetylcysteine (NAC) is an antioxidant and anti-inflammatory agent that is used in clinical practice [84]. In this report, a dendrimer conjugated with TPP and NAC was intravenously administered to rabbits with traumatic brain injury (TBI). The dendrimers successfully accumulated in mitochondria of the activated microglia at the site of the injury, contributing to the removal of ROS. A TPP-modified PLGA carrier encapsulating CoQ_10_ has been reported to prevent and treat neurocognitive impairment, which is a symptom of HIV and is a side effect associated with multidrug therapy [85]. Treatment of mouse or human neural progenitor cells (NPCs) with a TPP-modified PLGA carrier encapsulating CoQ_10_ before treatment with anti-HIV drugs resulted in a significant reduction in the amount of ROS. In addition, mitochondrial basal respiratory capacity and ATP production ability were significantly improved, suggesting that this strategy contributes to the neuroprotective effect.

#### 3.1.2. Validation of Anti-Oxidant Therapy by Mitochondrial Delivery of CoQ_10_ Using MITO-Porter System

**Figure 6:**
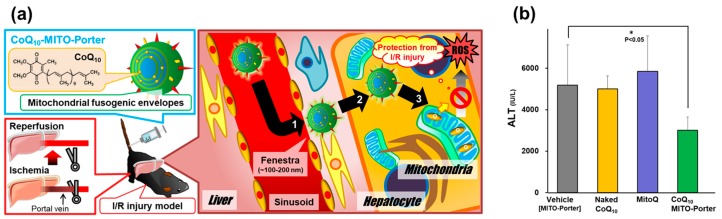
Overview of prevention of the hepatic ischemic reperfusion injury (IRI) by MITO-Porter system [86]. (**a**) Schematic image of antioxidant therapy by mitochondrial delivery of CoQ_10_ using MITO-Porter. (**b**) Evaluation of therapeutic effect of CoQ_10_-MITO-Porter. Serum alanine transaminase (ALT) activities were measured after the hepatic IRI. Data are represented as the mean ± S.D. (*n* = 4). Significant difference between vehicle and others was calculated by one-way ANOVA, followed by Bonferroni test. These figures are reproduced with permission from Elsevier.

Here, we describe our attempts to carry out anti-oxidant therapy by the mitochondrial delivery of CoQ_10_ using a MITO-Porter system. Figure 6a shows a schematic image of the prevention of hepatic ischemic reperfusion injury (IRI) by the mitochondrial delivery of CoQ_10_, an anti-oxidant chemical, using a MITO-Porter system. Under an ideal scenario, the MITO-Porter encapsulating CoQ_10_ reaches the liver tissue via systemic injection, and the carrier is then internalized by hepatocytes. In the cytosol, the carrier delivers CoQ_10_ to mitochondria via membrane fusion, resulting in the CoQ_10_ in mitochondria creating a pharmacological effect.

We quantified the accumulation of the radio isotope-labelled carrier in liver mitochondria after systemic injection. As a result, the MITO-Porter accumulated in the liver and liver mitochondria much more efficiently than negative control carriers [86]. These results indicate that the MITO-Porter was able to reach the liver via systemic injection, followed by mitochondrial targeting in the liver. To evaluate the therapeutic effect of the CoQ_10_-MITO-Porter, we administrated the CoQ_10_-MITO-Porter to mice, followed by inducing a hepatic IRI, then measured serum alanine transaminase (ALT) levels. In this experiment, it was expected that the delivery of CoQ_10_ to mitochondria in the liver of IRI induced mice would protect the liver from mitochondrial derived ROS, resulting in a decrease in ALT levels. As a result, we confirmed that the CoQ_10_-MITO-Porter resulted in a significant decrease in ALT-levels compared with naked CoQ_10_ and other carriers (Figure 6b) [86].

We are currently conducting clinical research directed toward trials to establish a viable mitochondrial therapeutic strategy. In order to start such a project, it is important to prepare nano particles with a high drug encapsulation rate. In view of this point, we investigated the encapsulation of CoQ_10_ in a MITO-Porter using various preparation methods, we found that the ethanol dilution method resulted in the efficient packaging CoQ_10_ in the carriers [87]. Furthermore, a technique for producing large amounts of particles with a high reproducibility is very important for practical applications. We previously attempted to manufacture a CoQ_10_-MITO-Porter using a microfluidic device, and we were able to reproducibly prepare the carriers on a large scale [88].

### 3.2. Cell Therapy to Reduce Oxidative Stress in Mitochondria

#### 3.2.1. Relationship between Mitochondrial Oxidative Stress and Cardiomyopathy

It has been reported that cardiomyocytes contain large amounts of mitochondria because of the high energy demand, which would result in higher oxidative stress in the myocardium. Once ischemia occurs in the heart, the blood supply to cardiac tissue stops and is then restored via a natural course or as the result of medical intervention. After reperfusion, many disorders such as heart attacks occur which can then lead to IRI. While the reperfusion of the ischemic heart is required for survival, it also massively exaggerates the extent of oxidative damage, the induction of cell death and inflammatory responses via ROS that are produced in mitochondria. Although mitochondrial ROS generation through IRI has been reported not only in the heart but also in other organs such as the brain, liver, kidney etc., it is generally thought to be a non-specific finding via reperfusion [89,90,91].

Murphy et al. recently reported that a comparative in vivo metabolomic analysis could be used to identify the reverse electron transport of mitochondrial electron transport that is responsible for the massive generation of mitochondrial ROS that occurs as the result of IRI [92]. DOX has been also reported to induce cardiotoxicity through redox cycling and ROS generation [93]. A number of clinical studies with ROS scavengers failed to identify a strategy for avoiding the cardiac damage due to DOX. Thus, the mechanism responsible for DOX -induced cardiac toxicity remains unclear. Zhang et al. first reported that the cardiomyocyte-specific deletion of *Top2b,* which encodes topoisomerase-IIb, could prevent cardiomyocytes from DNA double-strand breakage induced by DOX and transcriptome changes, which are responsible for the increased mitochondrial ROS production [94]. Ichikawa et al. also reported that DOX results in the selective accumulation of iron in mitochondria, revealing that DOX down-regulates ATP-binding cassette sub-family B member 8 (ABCB8) which is responsible for iron export from the mitochondria. These results strongly support the hypothesis that myocardial oxidative stress is due to iron-mediated ROS formation [95].

#### 3.2.2. Cell Therapeutic Strategy for Treating Cardiomyopathy

Cardiac progenitor cells (CPC) have been reported to have cardiac stemness and are currently used in cardiac regenerative therapies in clinical settings [96,97]. It has been reported that cardiac stem cell therapies hold some promise for dealing with myocardial oxidative stress due to systems such as IRI. They have also been reported to have some advantages in, not only the ischemic myocardium, but also in DOX-induced cardiomyopathy [98] and to confer resistance against oxidative stress in the damaged heart (Table 3). Though transplanting native cells would be effective, it would not be sufficiently efficient, without the additional manufacturing of artificial cell-tissue technology because of the exaggerated oxidative stress that becomes worse in the host-myocardium. Takehara et al. showed the usefulness of using CDC, whose lineage is similar to CPC, in transplantation in the ischemic myocardium, followed by the report from Aonuma et al. The findings revealed that cardiac stem cell transplants could be strengthened by the induction of an antioxidant gene like apurinic/apyrimidinic endonuclease/redox factor 1 (Ape1/Ref1) to overcome worsened oxidative stress [99,100].

The formation of mitochondrial ROS in the myocardium could play a major role in cardiac oxidative stress. Thus, to survive under exaggerated oxidative stress, new CPCs for stem cell therapy have been manufactured by delivering resveratrol, which has been reported to function as an antioxidant or a modifier of biogenesis in mitochondria, into mitochondria via the use of a MITO-Porter system [101,102]. The activated CPC, which are referred to herein as MITO cells, has been confirmed to have the necessary efficacy and efficiency in in vitro and in vivo DOX-induced cardiomyopathy models [101].

Cell transplantation combined with artificial technologies such as surgical techniques, sheet technology, or DDS technology, would be promising for the treatment of heart failure, rather than conventional cell transplants. We hope that this type of research will develop and be clinically used in the near future.

## 4. Conclusions

Various approaches have been taken to date to develop new and highly-efficient phototherapeutic methods for targeting mitochondria. The essential activities of mitochondria allow us not only to kill the cells but also activate them. As of this writing, there have been many reports focusing on oxidative stress, but studies directed to delivering functional molecules to mitochondria are not mature, and further research will be needed in the future. Even in such a situation, it seems that mitochondrial drug delivery with the goal of cancer therapy, i.e., killing cancer cells with ROS are a real possibility. On the other hand, mitochondrial DDS research that suppresses the induction of mitochondrial oxidative stress rather than killing cells involving conventional DDS such as a small ligand remains at the laboratory level. Therefore, we conclude that the development of DDS that secures the safety of mitochondria in target organs and delivers various molecules will greatly accelerate therapeutic strategies based on mitochondria ROS regulation. We wish to contribute to the quality of life for many patients with new treatments in terms of a DDS design.

## Figures and Tables

**Table 1:** Summary of mitochondrial targeting PDT and the PTT strategy for cancer therapy.

System Name	Irradiation Conditions	Evaluation	In Vitro EC_50_	Refs.
**Mitochondriotropic-Modified PS**				
**Verteporfin-TPP** TPP-modified verteporfin PS	Laser 690 nm	KB cells	*n.a.*	[25]
**Ir-P(ph)_3_** Iridium (III) complex as singlet oxygen PS conjugated with TPP	Xenon lamp 475 nm (39.6 J/cm^2^)	HeLa cells	*n.a.*	[28]
**Cationic octahedral molybdenum complex** Octahedral molybdenum complex PS conjugated with TPP	LED 460 nm (18 J/cm^2^)	HeLa cells	0.10 ± 0.02 μM	[33]
**Rh-SiPc** Si(IV)-phthalocyanine PS axially ligated with two rhodamine B molecules as mitochondriotropic	Tungsten lamp 500 nm (1–4 J/cm^2^)	HK-1	*n.a.*	[34]
**PpIX-PEG-(KLAKLAK)_2_** Conjugation of protoporphyrin IX (PpIX) PS with mitochondria-targeted (KLAKLAK)_2_ peptide	400–700 nm (4.75 J/cm^2^)	HeLa cells	30 mg/L	[35]
**IR700DX-6T** Chemical conjugation of IR700 PS with mitochondrial membrane TSPO ligand	LED 690 ± 20 nm (54 J/cm^2^)	MDA-MB-231 & MCF-7	*n.a.*	[36]
**Mitochondrial Targeted PS in DDS**				
**M-TPPa** pH-responsive mPEG-*b*-PDPA labeled with Cy7.5 as a nanocarrier system for TPP-conjugated pyropheophorbide-a	Laser 660 nm (15 J/cm^2^)	HO8910	*n.a.*	[29]
**HA-IR-Pyr** Micellar aggregate of mitochondrial targeting PS, IR-Pyr, and Hyaluronic acid as cancer selective delivery agent	Laser 808 nm (36 J/cm^2^)	HeLa cells & MDA-MB-231	5–7 μM	[38]
**NGO-PEG-FA/MitoTPP** Mitochondrial targeting PS (MitoTPP) incorporated into PEGylated-nanographene (NGO) functionalized with tumor targeting folic acid	LED 650 nm (18 J/cm^2^)	HeLa cells, L929, & A549	*n.a.*	[39]
**PS@chol-BSA NPs** TPP-modified pheophorbide-a as the mitochondria selective PS encapsulated with folate-cholesteryl bovine serum albumin as the tumor-selective nanocarrier	Laser 671 ± 1 nm (1.5 J/cm^2^)	U87MG	0.81 μg/mL	[40]
**Mitochondriotropic-Modified DDS**				
**TPP-IR780/Ce6-TNS** Chlorin e6 PS and IR780 incorporated into TPP-modified lipid nanoparticles. IR780 acts as PTT agent and to control PDT process	Laser 808 nm (IR780) followed by 660 nm (Chlorin e6)	HeLa cells	*n.a.*	[41]
**UCNPs@TiO_2_-TPP** TPP-modified TiO_2_-coated UCNPs. UCNPs harvest NIR light and emit UV light to activate TiO_2_ for ROS production	Laser 980 nm (90-450 J/cm^2^)	MCF-7	*n.a.*	[30]
**UCNP-GQD/TRITC** Hybrid nanoparticles of UCNP and ^1^O_2_ generator of graphene quantum dot (GQD) with TRITC surface modification as mitochondriotropic	Laser 980 nm (720 J/cm^2^)	4T1 cells	*n.a.*	[45]
**TAT-Ppa-UCNPs** PEGylated polymer-coated UCNP carrying pyropheophorbide a (Ppa) PS with TAT surface modification	Laser 808 nm (1800 J/cm^2^)	HeLa cells	*n.a.*	[46]
**MITO-Porter System**				
**rTPA-MITO-Porter** An NIR PS, rTPA, delivered by MITO-Porter system via macropinocytosis followed by electrostatic interaction and fusion with mitochondrial membrane	Xenon lamp 700 ± 6 nm (12.8 J/cm^2^ & 20.6 J/cm^2^)	HeLa cells & SAS cells	0.16 – 0.41 μM (0.26–0.64 μg/mL)	[49]
**PTT Reagents**				
**Mito-CCy** Cryptocyanine-based PTT reagent with mitochondrial targeting moiety TPP	Laser 730 nm (1.4 kJ/cm^2^)	HeLa cells	*n.a.*	[50]
**TPP-Au** Gold nanoparticles tethered with mitochondrial targeting moiety TPP	Laser 808 nm (1.4 kJ/cm^2^)	HeLa cells & COS7 cells	7.0–8.5 μg/mL	[51]
**T-3-BP-AuNP** Gold nanoparticles tethered with mitochondrial targeting moiety TPP and 3-bromopyruvate which is a decoupling reagent of the respiration	Laser 660 nm (1.2 J)	PC3 cells	10 μg/mL	[52]

**Table 2:** Summary of mitochondrial antioxidants delivery using TPP system.

Name	Cargo	Model/Administration Route	Nanoparticle	Outcomes	Refs.
MitoQ	Coenzyme Q_10_	Neuronal HT22 cells and Mouse embryonic fibroblasts	—	Decreasing oxidative stress	Jelinek A. et al. (2018) [77]
Mito-TEMPO	2,2,6,6-tetramethylpiperidine 1-oxyl	APAP-induced liver injury mouse/Intraperitoneal injection	—	Attenuating the mitochondrial oxidant stress and preventing peroxynitrite formation and the subsequent mitochondrial dysfunction	Du K. et al. (2017) [78]
MitoC	Ascorbate	Rat liver mitochondria	—	Preventing mitochondrial oxidative damage	Finichiu PG. et al. (2015) [79]
Mito-Apo	Apocynin	Immortalized rat mesencephalic cells (N27), LUHMES cells	Brain targeting nanoparticle (CPH:SA = 20/80) with FA	Protection against oxidative stress-induced mitochondrial dysfunction and neuronal damage in a dopaminergic neuronal cells.	Brenza TM. et al. (2017) [81]
MitoPBN	PBN	L02 cells and 293T cells Diabetic mouse model/Intraperitoneal injection	Liver targeting nanoparticle (Cholesterol:lecothin = 1/2)	Alleviating ROS-induced mitochondrial dysfunction	Wu M. et al. (2019) [82]

APAP, Acetaminophen; Apocynin, 4-hydroxy-3-methoxyacetophenone; CPH, 1,6 bis(p-carboxyphenoxy)hexane; FA, folic acid; ISL, injectable soybean lecithin; LUHMES, Lund human mesencephalic; PBN, phenyl tert-butylnitrone; PLGA, poly (D,L-lactide-co-glycolide); SA, sebacic acid; TPP, Triphenylphosphonium.

**Table 3:** Cell therapeutic strategy for treating cardiomyopathy.

Disease Model	Type of Cell Source	Cell Modification	Method of Transplant	Main Outcome	Refs.
IRI, pig	Human CDC	None	Cell sheet	Reduced infarct size and improved EF	Takehara N. et al. (2008) [99]
IRI, mouse	Mouse CPC	Overexpression of APE1/REF1 gene	Intramyocardial injection	Attenuation of fibrosis and improved EF	Aonuma T. et al. (2016) [100]
DOX-CM, mouse	Mouse CPC	Delivery of resveratrol into mitochondria	Intramyocardial injection	Reduced oxidative stress of myocardium and longer survival time	Abe J. et al. (2018) [101]

IRI, Ischemic reperfusion injury; DOX-CM, Doxorubicin-induced cardiomyopathy; CDC, Cardiac derived sphere cell; CPC, Cardiac progenitor cell; APE1/REF1, Apurinic/apyrimidinic endonuclease/redox factor 1; EF, Ejection fraction.
